# Habitat selection by vulnerable golden bandicoots in the arid zone

**DOI:** 10.1002/ece3.7875

**Published:** 2021-07-08

**Authors:** Cheryl A. Lohr, Kristen Nilsson, Colleen Sims, Judy Dunlop, Michael T. Lohr

**Affiliations:** ^1^ Department of Biodiversity Conservation and Attractions Biodiversity Conservation Science Kensington Western Australia Australia; ^2^ Phoenix Environmental Sciences Osborne Park Western Australia Australia; ^3^ School of Science, Edith Cowan University, 100 Joondalup Drive Joondalup WA 6027 Australia

**Keywords:** Australia, Indigenous Protected Area, marsupial, restoration, spinifex grassland, translocation

## Abstract

In 2010, vulnerable golden bandicoots (*Isoodon auratus*) were translocated from Barrow Island, Western Australia, to a mainland predator‐free enclosure on the Matuwa Indigenous Protected Area. Golden bandicoots were once widespread throughout a variety of arid and semiarid habitats of central and northern Australia. Like many small‐to‐medium‐sized marsupials, the species has severely declined since colonization and has been reduced to only four remnant natural populations. Between 2010 and 2020, the reintroduced population of golden bandicoots on Matuwa was monitored via capture–mark–recapture data collection, which was used in spatially explicit capture–recapture analysis to monitor their abundance over time. In 2014, we used VHF transmitters to examine the home range and habitat selection of 20 golden bandicoots in the enclosure over a six‐week period. We used compositional analysis to compare the use of four habitat types. Golden bandicoot abundance in the enclosure slowly increased between 2010 and 2014 and has since plateaued at approximately one quarter of the density observed in the founding population on Barrow Island. The population may have plateaued because some bandicoots escape through the fence. Golden bandicoots used habitats dominated by scattered shrubland with spinifex grass more than expected given the habitat's availability. Nocturnal foraging range was influenced by sex and trapping location, whereas diurnal refuge habitat, which was typically under a spinifex hummock with minimal overstory vegetation, was consistent across sex and trapping location. Our work suggests that diurnal refuge habitat may be an important factor for the success of proposed translocations of golden bandicoots.

## Graphical abstract

Historically, the golden bandicoot (*Isoodon auratus*) spanned approximately 2,000 km across northern Australia. Today, only four remnant natural populations remain. Here, we describe the successful reintroduction of the locally extinct golden bandicoot to an ex‐pastoral lease in the rangelands of Western Australia. Further, we describe habitat selection by golden bandicoots and provide information crucial to the design of future translocations proposed for the species.

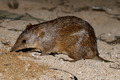

## 1 INTRODUCTION

**FIGURE 1:**
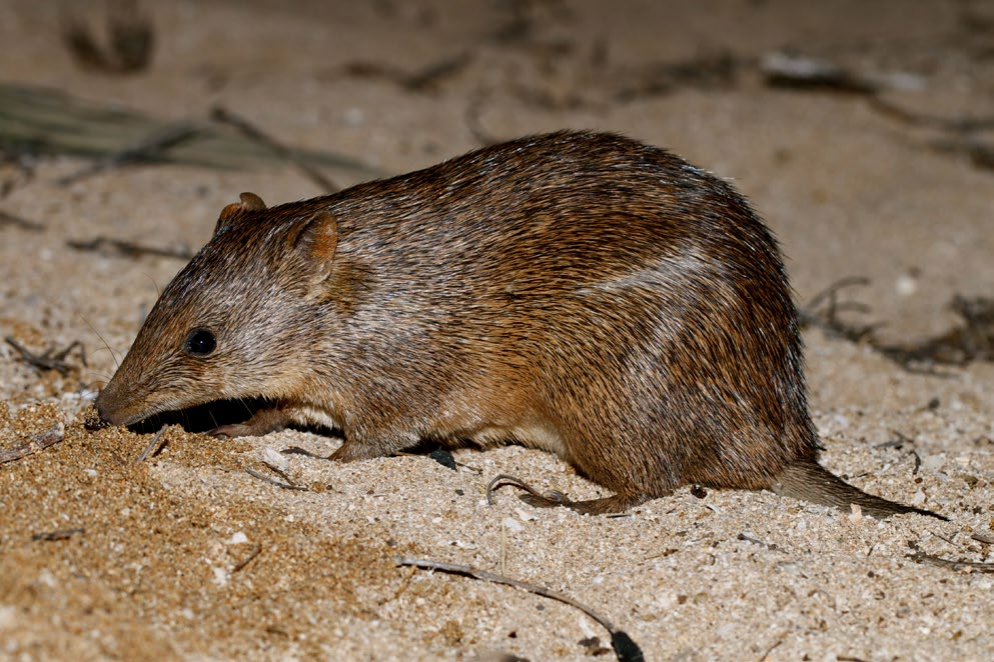
Golden bandicoot (*Isoodon auratus*). Photo credit, Judy Dunlop

Since European settlement, Australia's terrestrial mammal fauna has suffered a severe and continued decline (Burbidge et al., 2009; Geyle et al., 2018) and 30 of 273 Australian endemic mammal species have become extinct (Woinarski et al., 2015). Arid zone mammal species, within the Critical Weight Range (CWR) of 35 g–5.5 kg have suffered disproportionately in the decline (McKenzie et al., 2007). The golden bandicoot (*Isoodon auratus*; Figure 1) have declined from a historic range that spanned approximately 2,000 km across northern Australia from central Western Australia to western Queensland to four remnant natural populations in the northwest Kimberley, Marchinbar Island in the Northern Territory, four islands along Kimberley coast (Gibson & McKenzie, 2012) and two islands along Pilbara coast (Burbidge & Woinarski, 2016).

Globally, conservation translocations are used to establish new populations to reduce the risk of extinction for threatened species (IUCN/SSC, 2013). Translocations to closed systems such as enclosures or islands are commonly used for conservation of fauna species that are particularly susceptible to predation by introduced species such as feral cats (*Felis catus*) or red foxes (*Vulpes vulpes*) (Ringma et al., 2018). Accumulative conservation evidence, with a total of twenty‐four studies from around the world, evaluated the effects of releasing translocated mammals into fenced areas and found that the method improves the likelihood of success by increasing reproductive success, survival, and body condition of the species being translocated (Littlewood et al., 2020). However, fenced populations may experience issues with overpopulation and competition due to restricted dispersal (Moseby et al., 2018; Saifuddin et al., 2017); loss of predator awareness (Rowell et al., 2020); or inbreeding depression (Ottewell et al., 2014; Rick et al., 2019).

Instances of poor persistence of species in closed systems are likely to stem from incomplete knowledge of the biology and ecology of the species (Rayner et al., n.d.). Many threatened species are described as data deficient because either they are cryptic and difficult to research, or their populations declined before science could adequately document the ecological characteristics of the species. In these situations, conservation translocations are often experimental trials that may be used to test the efficacy of translocation techniques (Clarke et al., 2002; Priddel & Carlile, 2001) or identify resource requirements of the species (Stannard et al., 2010) and suitability of the translocation site (Bester & Rusten, 2009). Successful translocations to fenced enclosures subsequently provide an opportunity to research the biology and ecology of threatened species in a limited but potentially diverse array of habitats present in the species original distribution. Knowledge gained in these environments can provide valuable insights, which may benefit future translocations.

To date, there have been few studies on habitat selection by golden bandicoots. Previous studies have occurred in subtropical regions of the Kimberley (*n* = 8 bandicoots, t = 5 days) (Graham, 1996) and Marchinbar Island in the Northern Territory (*n* = 12 bandicoots, t < 21 days), where golden bandicoots used a range of daytime shelters in a relatively stable 10–35 ha home range (Southgate et al., 1996), but have used few individuals and have limited applicability to desert or rangeland habitat. Short and Turner (1994) investigated the importance of habitat heterogeneity for a range of marsupial species (including the golden bandicoots) on Barrow Island and concluded that the absence of introduced predators and herbivores had a greater impact on the abundance and distribution of bandicoots within the spinifex grassland habitat than any vegetation mosaic or disturbance characteristic.

In this study, we examine the abundance, home range, and habitat use of a translocated population of golden bandicoots in a mainland, arid zone, introduced predator‐free, fenced enclosure on the Matuwa Indigenous Protected Area (IPA) in Western Australia (henceforth “Matuwa”). Over time, we expected the abundance and density of golden bandicoots within the enclosure to mimic or surpass the density recorded on Barrow Island (Teale, 2013). Home‐range data were collected in 2014, 4 years after their reintroduction from Barrow Island (2021 n.d.). We mapped the broad vegetation categories within the enclosure using satellite imagery and ground truthing and then used compositional analyses from radiotelemetry data to infer habitat selection at the second and third order (Johnson, 1980). Second‐order selection is the individual's home range within their geographic range, and third‐order selection is their habitat use within their home range (Johnson, 1980). We expected bandicoots to select vegetation with an understory of hummock/spinifex grasses (*Triodia* sp.) for shelter and protection, which is similar to habitat used by the source population on Barrow Island (Bradshaw et al., 1994), rather than open mulga (*Acacia* sp.) woodlands. Additionally, we expected home‐range characteristics to differ between the sexes, specifically for male home range to be larger due to forays into adjoining territories as seen in Kimberley populations (Graham, 1996).

## 2 METHODS

### 2.1 Study location

**FIGURE 2:**
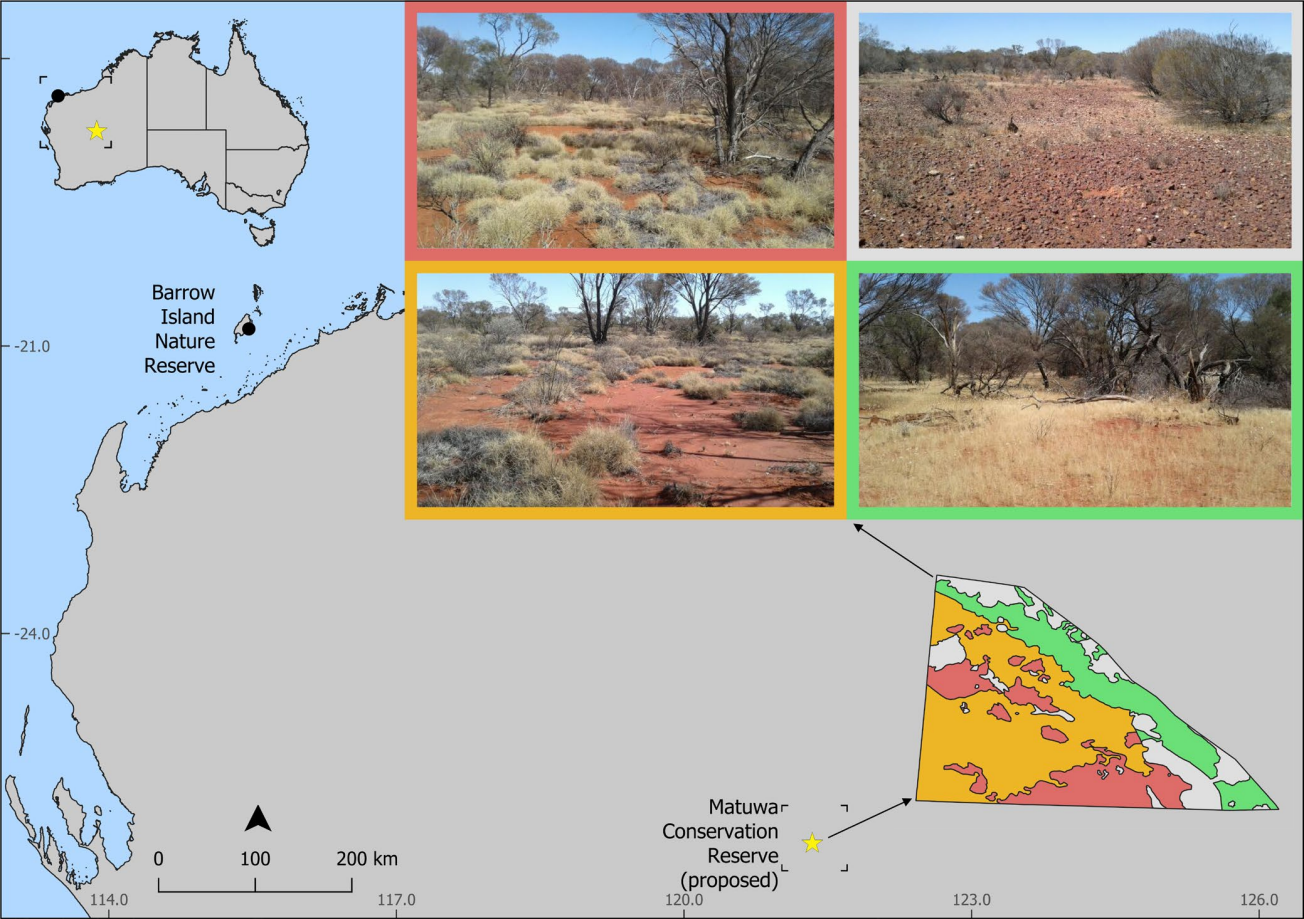
Map of Australia showing the location of source (Barrow Island) and translocated population (Matuwa fenced reserve enclosure), and the four broad vegetation types occurring within the enclosure. Orange symbolizes scattered mixed shrubland over spinifex grass, green symbolizes dense mulga with tuft grass, red symbolizes dense shrubland with spinifex grass, and light gray symbolizes bare understory

Matuwa (244,000 ha) lies in central Western Australia (−26.1986; 121.3598) and straddles the Murchison and Gascoyne Interim Biogeographic Regionalisation for Australia (IBRA) regions (Department of Agriculture Water and the Environment, 2020; Figure 2). It contains at least 20 different land systems and vegetation types such as hummock grasslands, shrublands, or low woodland with mulga. This diverse habitat supports a high diversity of flora and fauna, with 480 vascular plant species and 220 vertebrate species occurring on the property (Baynes, 2006; Chapman & Burrows, 2015; Coate, 2010; Department of the Environment Water Heritage and the Arts, 2009; Rabosky et al., 2011). Matuwa has an arid climate with an average monthly diurnal temperature of 30°C in summer and 13°C in winter. The mean annual rainfall is 250 mm, which primarily occurs in the summer months due to remnant tropical low‐pressure systems.

**FIGURE 3:**
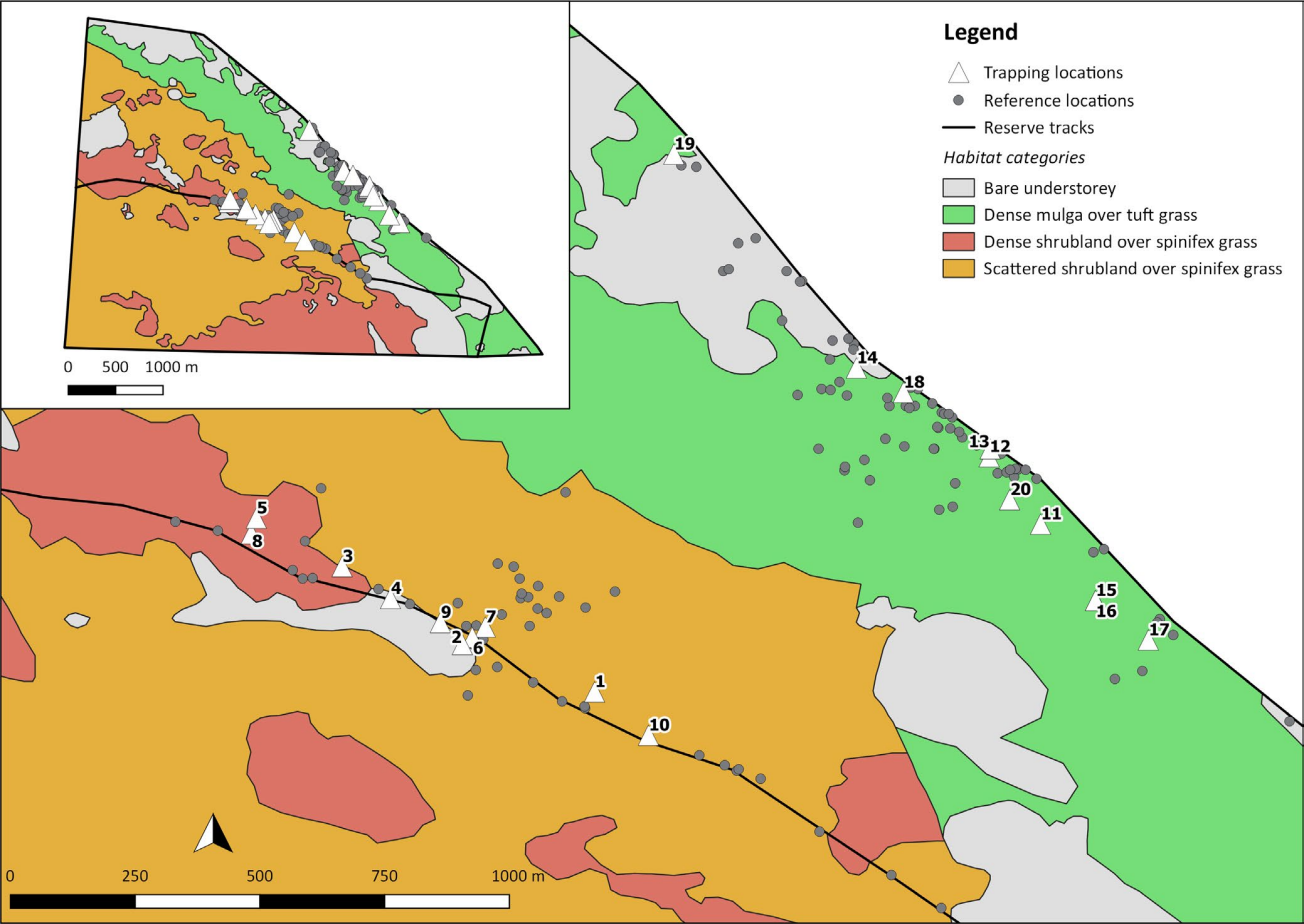
Habitat map of the Matuwa fenced enclosure plus numbered trap locations of the 20 golden bandicoots that were VHF tracked, and reference locations of surveyors for detecting transmitter signals

The enclosure on Matuwa (26°13’S, 121°33’E) was constructed in 2009/10 (Bode et al., 2012) and encompasses approximately 1,100 ha of mixed habitats, including spinifex grassland (mainly *Triodia basedowii*) under acacia, and mallee eucalypt shrub overstory in the Murchison bioregion; and mulga (*Acacia aneura*) woodland rather than sparse to very sparse understory of tufted grasses (*Aristida* sp.) in the Gascoyne bioregion (Figures 2 and 3).

### 2.2 Study species

Golden bandicoots are listed nationally as vulnerable under the Environment Protection and Biodiversity Conservation Act 1999 (Burbidge & Woinarski, 2016). Formerly widespread across Australia's arid and semiarid zones, by 2010, golden bandicoots, which are primarily insectivores (Radford, 2012), were restricted to Barrow and Middle islands (WA Pilbara) (Dunlop & Morris, 2018), Augustus, Lachlan, Storr, and Uwins islands (WA Kimberley) (Gibson & McKenzie, 2012), Marchinbar Island (NT Arnhem Land) (Southgate et al., 1996), and high‐rainfall areas of the northwestern Kimberley between Yampi Peninsula and Mitchell Plateau (Palmer et al., 2003).

### 2.3 Translocation

In 2010, as part of the Environmental Offset Conditions attempting to ameliorate the impact of the Gorgon Gas Development on Barrow Island, golden bandicoots were translocated from Barrow Island, Western Australia, to several new sites in Western Australia, including Hermite Island in the Montebello Group, Doole Island in Exmouth Gulf, and an enclosure on Matuwa (2021 n.d.; Western Australia Government, 2003). This translocation to Matuwa involved a total of 160 (78 female, 82 male) bandicoots transferred via car, helicopter, and fixed‐wing aircraft and released within 24 hr of capture (Dunlop, 2015). Boodies (*Bettongia lesueur*), mala (*Lagorchestes hirsutus*), and brushtail possums (*Trichosurus vulpecula*) have also been successfully translocated into the fenced enclosure (Lohr, 2019). Small mammal species can pass through the fence, including brush‐tailed mulgara (*Dasycercus blythi*), spinifex‐hopping mice (*Notomys alexis*), and subadult golden bandicoots.

### 2.4 Abundance estimates

The abundance of golden bandicoots in the fenced enclosure is monitored at least annually, through capture–mark–recapture (CMR) surveys. Intervals between trapping sessions are irregular, with six sessions in 2010 subsiding to annual sessions by 2015. We have analyzed all data collected between 2010 and 2020 over a total of 24 primary trapping sessions, with a variety of trap layouts from single traps spaced every 200 m along access roads to high‐density clusters of traps within 50 m of active boodie warrens. We excluded any trapping sessions that specifically targeted boodies with trap layouts that only included traps near warren entrances and provided limited opportunity for capture of other species.

Analysis of trapping data was conducted via the open population, spatially explicit capture–recapture analysis in the R package “openCR” (Efford, 2019) with the fenced area as a closed survey mask and multicapture traps. We used the JSSAsecrD modeling framework because we were interested in estimates of the abundance of bandicoots. We tested models that allowed the four parameters within JSSAsecrD (*sigma*, *lambda0*, *phi*, and *D*) to vary among sessions (*t*) and models that held *sigma* and *lambda0* constant while allowing *phi* and *D* to vary. We analyzed datasets for male and female bandicoots separately as well as a full combined dataset. Sex was not included as a variable in the combined dataset. The estimate of density (*D*) was converted to an estimated abundance by multiplying values by the area of the mask (1,120 ha).

Due to frequent trap interference by boodies, which diminishes our ability to accurately estimate the number of golden bandicoots inside the enclosure, trap files were modified, with any traps that captured a boodie being listed as inactive traps on that occasion (henceforth “subset CMR”). We used the moving.fit function within openCR to analyze blocks of five consecutive primary trapping sessions. Standard error margins are derived from variation in outputs of the moving.fit function for each session.

### 2.5 Habitat mapping

Vegetation within the enclosure was delineated using satellite imagery in conjunction with ground truthing. Four broad vegetation classifications (Figure 3), and their proportions, were demarcated as follows: scattered mixed shrubland with spinifex grass (43.8% of fenced enclosure), dense shrubland with spinifex grass (20.5%), dense mulga with tuft grass (19.6%), and bare understory (16.1%). Mulga overstory species were most commonly *Acacia aneura*.

### 2.6 Radiotracking

Twenty‐seven golden bandicoots were trapped using small cage traps (20 cm × 20 cm × 56 cm, Sheffield Wire Co. Welshpool), with a rolled oats and peanut butter bait from the 12th to the 14th of August 2014. Trapping occurred along the central and northern track of the enclosure (Figure 3). The central and northern trapping lines were designed to capture habitat heterogeneity across the enclosure with bare understory or dense mulga with tuft grass dominating the northern region, and scattered shrubland with spinifex grass dominating the central region (Figure 3). Twenty adult animals (6 females and 14 males, 10 from each trap line) were fitted with Titley TX GP1‐1/3N, PIC 3.0V 2‐stage VHF transmitters, with 60 ppm pulse rate, 12 hr mortality switch (changing to 80 ppm), with 250‐mm antenna and 60‐day battery life, on an 8‐mm wide soft leather collar, weighing approximately 9 g, at their point of capture and immediately released. Radio signals were detected from transmitters using the Sirtrack Ultra receivers. Radiotracking began one day after an individual was radio‐collared and released and ended on 22 September 2014 (period of 5.6 weeks). Five individuals (GB 3, 10, 14, 19, and 20; Table S2) were tracked for less than 50% of this period, with detections lost between weeks 1–3. Two golden bandicoots (#3 and #14) were found dead, on the 1st of September and the 28th of August, respectively, with raptor predation the suspected cause of death.

During the day, telemetry positions of golden bandicoots were determined by homing directly to their refuge location between the hours of 12 p.m. and 5 p.m. (diurnal locations). Soil type, plant species, and/or type of refuge, and vegetation characteristics of the surrounding landscape, were all recorded. During the night, three signal bearings, recorded at three different receiving locations, within five minutes of each other were used to determine an individual's location between the hours of 7 p.m. and 12 a.m. (nocturnal locations). Surveyors used reference points, which were along tracks approximately 200 m apart as initial tracking locations. The reference location, bearing of the transmitter signal, and strength of the signal were all recorded. Radio collars were removed from animals from the 24th to the 27th of September 2014.

### 2.7 Home range

Using LOAS software (Ecological Software Solutions, 2000), we triangulated a golden bandicoot's location from signal bearings using the maximum likelihood estimator (≥3 bearings) or the Best Biangulation (2 bearings) as a backup method. A total of 586 groups of bearings were input into LOAS. LOAS rejected 57 of these points using a priori set of rules, and a further 53 were manually removed as they fell outside the boundary of the enclosure. As a result, a total of 110 radiolocations were removed from the analysis. This left 475 telemetry locations for the nocturnal period. Of these 475 locations, 282 (59%) were triangulations and 193 (41%) were biangulations. For each of the 20 bandicoots, number of telemetry positions ranged from 8 to 48, with 75% having ≥30 telemetry positions.

Using BIOTAS software (Ecological Software Solutions, 2000), we estimated the home ranges of animals using the 475 nocturnal radiolocations, and 228 identified refuge locations. We calculated a minimum convex polygon (MCP), and the 95% fixed kernel density estimators (KDE), with least‐squares cross validation, which is relatively robust to small sample sizes (Gredzens et al., 2014; Seaman & Powell, 1996). We chose to report the MCP results but exclude them from subsequent analysis due to the limitations of this method (Börger et al., 2006). Estimated home ranges were then cropped to the boundary of the enclosure. Differences in home‐range size between sexes, temporal periods, and trapping locations were tested using an independent *t* test assuming unequal variances. Additionally, due to our small sample size, we tested if the exclusion of biangulations from our dataset would produce significantly different home ranges to those calculated using both biangulations and triangulations with independent *t* test assuming unequal variances.

### 2.8 Habitat selection

We examined habitat selection at two scales, second order and third order, using 95% KDE. We imported telemetry locations and home‐range extents into QGIS v3.14 and overlayed them with the habitat vegetation map to calculate proportions utilized by each animal. Four vegetation categories were identified within the enclosure and used in analyses: scattered shrubland with spinifex grass, dense shrubland with spinifex grass, dense mulga with tuft grass, and bare understory (Figure 3). When a habitat type was available but not utilized, a small positive value (<0.001) less than the smallest proportion recorded for an individual was used in place of zero (Aebischer et al., 1993).

Second‐order and third‐order habitat selection values were analyzed separately for the two temporal periods (diurnal and nocturnal) and two trapping locations. Data were analyzed via linear mixed effects models with bandicoot ID as a random factor via the package lme4 (Bates et al., 2014). To assess whether relative habitat use was nonrandom (significantly different to zero), we set a dummy habitat variable with a relative use value of zero as the reference variable.

### 2.9 Radiotracking error

We performed a post hoc assessment on our triangulation data to evaluate the relationship between survey parameters and triangulation covariance. Survey parameters included the minimum, maximum, and average distance between receivers, the minimum, maximum, and average distance between receiver and the estimated signal, and the minimum, maximum, and total bearing angle of the estimated signal. We performed a linear regression using R (R Core Team, 2018), guided by AIC models in package AICcmodavg 2.2‐2 (Mazerolle, 2020), to detect significant relationships between our predictor variables and triangulation covariance. Covariance values ranged from 0.02 to 1.32 × 10^7^. We removed 146 datapoints considered outliers to normalize our data.

## 3 RESULTS

### 3.1 Abundance estimates

To estimate the abundance of golden bandicoots inside the fenced enclosure at Matuwa, we ran 12 model simulations using openCR consisting of a combinations of two datasets (complete CMR or subset CMR), two model formulations (*Global* *= sigma ~ t, lambda0 ~ t, phi ~ t, D ~ t; or PhiD = sigma ~ 1, lambda0 ~ 1, phi ~ t, D ~ t*), and three groups of bandicoots (females, males, or both sexes combined).

The *Global* model and *PhiD* model produced markedly different results. Estimates of bandicoot abundance in the *Global* model were approximately 20% larger than estimates in the *PhiD* model and fluctuated widely (Figure S1), suggesting the *Global* model is overparameterized. We rejected this model in favor of the reduced *PhiD* model. Removing traps that captured alternative species from the CMR dataset increased the estimated abundance of male bandicoots by the *PhiD* model by 3%–4% but resulted in minimal change in estimates of female abundance (Table 1).

**TABLE 1:** Average percent change in abundance estimates for golden bandicoots within the fenced enclosure at Matuwa when any traps that captured an alternative species were removed from the dataset for the global model (sigma ~ t, lambda0 ~ t, phi ~ t, D ~ t) and the *PhiD* model (sigma ~ 1, lambda0 ~ 1, phi ~ t, D ~ t)

Sex	Model	Δ All captures → subset CMR (%)
Both	*PhiD*	4.03
Female	*PhiD*	−1.31
Male	*PhiD*	3.19

**FIGURE 4:**
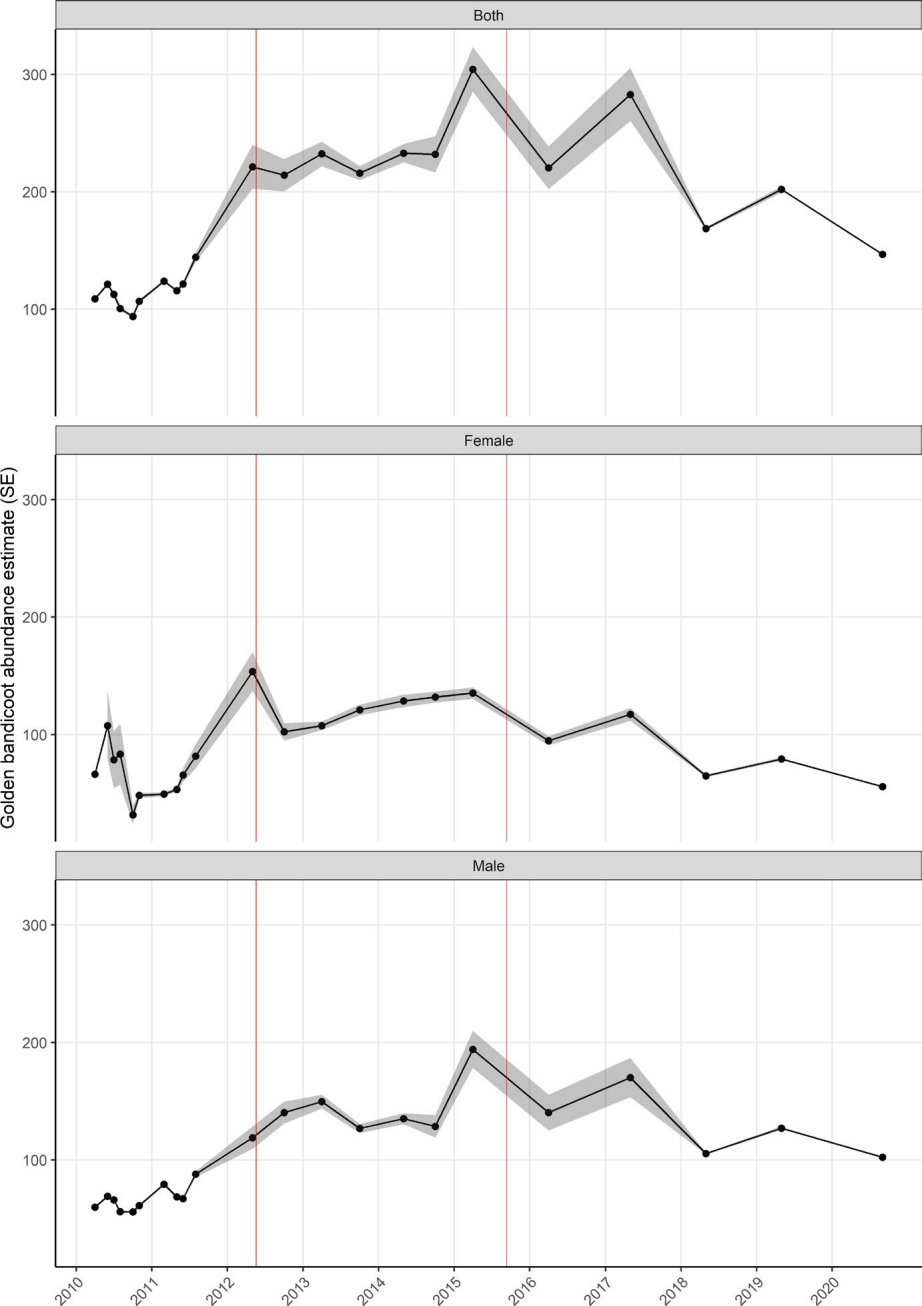
Estimates of the abundance of golden bandicoots within the fenced enclosure at Matuwa from subset CMR data that excluded any traps that captured alternative species and *PhiD* models that held sigma and lambda0 constant while allowing phi and D to vary. Results of other models are available in Figure S1. Red vertical lines depict the translocation of 49 and 93 bandicoots out of the fenced enclosure onto unfenced areas of Matuwa

In January 2010, 160 golden bandicoots were translocated from Barrow Island into the fenced enclosure at Matuwa. Our results (*PhiD* model, subset CMR) suggest that in the 10 months following the translocation the number of bandicoots declined to a low of 93 (*SE* = 11; 95% CI = 75–118) bandicoots in October 2010 before the population became established and started to increase (Figure 4). It was not until August 2011 that our estimates of abundance approach 160 bandicoots (*SE* = 18; 95% CI = 129–198). The population peaked in April 2015 with an average estimate of 304 (*SE* = 66; 95% CI = 285–323) and a maximum estimate of 393 (*SE* = 49; 95% CI = 297–533).

In 2012 and 2015, 49 and 93 golden bandicoots were removed from the fenced enclosure and translocated to unfenced areas on Matuwa (Figure 4). In the results of the *PhiD* model built using the subset CMR dataset (Figure 4), the number of bandicoots removed correlates closely with the change in the estimated abundance of bandicoots during surveys either side of the translocation. This suggests that the *PhiD* model provides reliable estimates of bandicoot abundance over time.

By May 2017, the bandicoot population inside the enclosure had recovered from the removal of 93 individuals 20 months earlier, returning to an average estimate of abundance of 282 golden bandicoots (*SE* = 67; 95% CI = 260–305). Subsequently, in 2018, the estimated abundance of golden bandicoots was particularly difficult to obtain and potentially suppressed by a dramatic increase in the number of boodies interfering with traps (Treloar et al., n.d.). In 2019, Matuwa recorded below average rainfall with only 69.5 mm of rain in 12 months (average 262 mm) that may have reduced the probability of survival for some individuals. Analysis suggested that survival (phi) dropped from 0.38 to 0.006, but survival estimates in 2018 were confounded by boodie interference and should be treated with caution. While the number of confounding variables makes it difficult to confirm the precise abundance, we can conclude that the population of golden bandicoots within the fenced enclosure at Matuwa is established and relatively stable with a maximum estimated density of 0.35 bandicoots per hectare.

### 3.2 Home range

In total, we collected 703 telemetry positions across the 20 bandicoots within the Matuwa enclosure. The comparison between the total dataset and the dataset excluding biangulations showed that the 95% KDE did not differ significantly between the sets of data (*p* = .50) (Table 2). Less accurate biangulations did affect estimates of MCP. Therefore, the total dataset and 95% KDE estimates were used for subsequent analyses.

**TABLE 2:** Comparison of mean home range and standard error between the total dataset and the dataset excluding biangulations

Dataset	Telemetry positions	95% KDE ha (*SE*)	MCP ha (*SE*)
Total data	703	18.08 (±5.66)	54.72 (±9.71)
Data with biangulations excluded	510	18.03 (±5.33)	27.22 (±4.70)

There were 228 refuge locations identified during the diurnal period, and 475 active locations identified using triangulation during the nocturnal period. Home‐range sizes for the nocturnal period were larger than those in the diurnal period (*p* = 2.43 × 10^−4^). The mean 95% KDE was 18.08 ha (±5.66), whereas mean MCP was 54.72 ha. KDE were considerably smaller than MCP due to the repeated use of diurnal refuge sites. Male bandicoots had 26%–45% larger home ranges than females across temporal periods (Table 3), although values were not statistically significant (*p* = .09). When only nocturnal foraging range was analyzed, males had a significantly larger home range than females (*p* = .02). There was no significant difference in diurnal sheltering range between sexes (*p* = .10).

**TABLE 3:** Summary of mean home ranges for golden bandicoots calculated using MCP and fixed 95% kernel density estimates in the Matuwa enclosure

Sex	Temporal Period	*n*	Mean 95% KDE (ha) (*SE*)	MCP ha (*SE*)
Male	Diurnal	152	8.45 (±4.01)	3.09 (±0.65)
	Nocturnal	312	84.77 (±19.17)	46.68 (±9.02)
Subtotal		464	21.64 (±7.89)	48.64 (±8.62)
Female	Diurnal	76	2.97 (±0.45)	4.21 (±3.42)
	Nocturnal	163	34.64 (±12.28)	68.19 (±26.26)
Subtotal		239	9.77 (±2.96)	68.90 (±26.17)
Central track	Diurnal	118	5.03 (±1.06)	2.62 (±0.86)
	Nocturnal	269	46.90 (±12.05)	53.63 (±17.01)
Subtotal		387	14.08 (±2.41)	55.05 (±16.70)
Northern track	Diurnal	110	8.79 (±5.66)	4.08 (±1.67)
	Nocturnal	206	92.56 (±25.49)	52.63 (±11.24)
Subtotal		316	22.08 (±11.23)	54.39 (±10.90)
Total		703	18.08 (±5.66)	54.72 (±9.71)

Bandicoots trapped along the northern track (henceforth “northern bandicoots”) showed larger home ranges to bandicoots trapped along the central track (henceforth “central bandicoots”) (Table 3). Values were not statistically significant across temporal periods but approached significance when only nocturnal foraging range was analyzed (*p* = .06). These results indicate variation in activity between sexes and between animals trapped along the central track and the northern track, in the nocturnal foraging range but not within the diurnal sheltering range.

### 3.3 Habitat selection

Second‐order selection by bandicoots (home‐range composition relative to availability within the enclosure) was nonrandom (*F*_(10, 89)_ = 7.05; *p* = 4.56^−8^; Adj *R* ^2^ = .38). Results of linear mixed effects models suggest that across all bandicoots, mulga with tuft grass was used significantly less than other habitat types (Table S1; coefficient = −2.69; *p* = .03). Sex and trapping location did not independently affect second‐order habitat selection (*p* = 1.00), but there was a significant interaction between habitat type and trapping location with dense shrubland with spinifex being used less than random by bandicoots trapped on the northern track (coefficient = −4.46; *p* = .009) and mulga with tuft grass being used significantly more than random (coefficient = 5.82; *p* = 8.50^−4^) by bandicoots trapped on the northern track. The three‐way interaction was not significant and was removed from the model. Bandicoot ID as a random effect accounted for very little variance (5.70^−21^) with residual variance equal to 7.11. Analysis of third‐order selection data via linear mixed effects models produced very similar results regarding residuals suggesting our methods sufficiently mitigated sources of autocorrelation.

**FIGURE 5:**
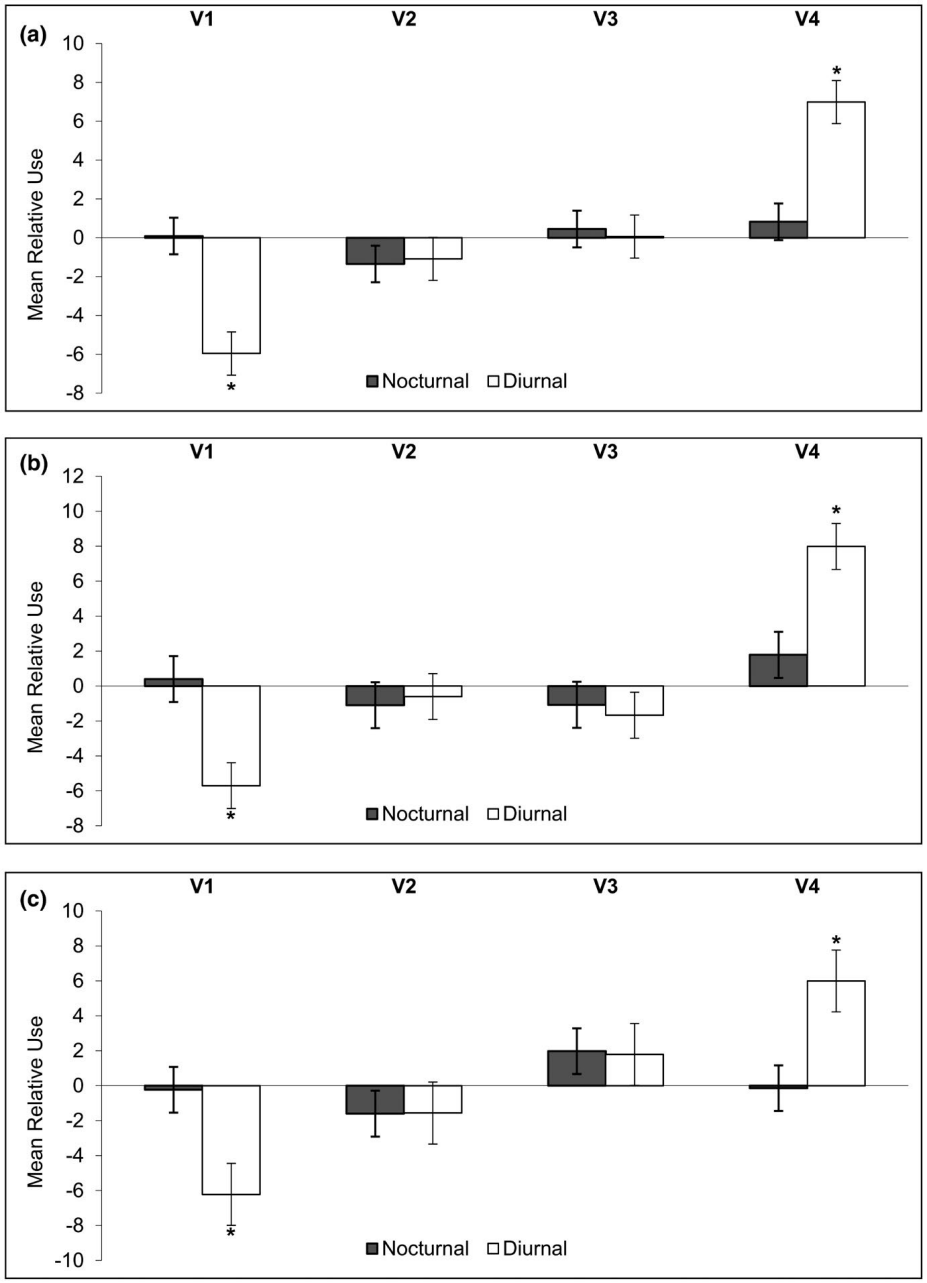
Mean third‐order habitat selection (using the 95% KDE) of golden bandicoots during their nocturnal and diurnal period for four habitat types; V1—bare understory, V2—dense shrubland with spinifex grass, V3—dense mulga with tuft grass, V4—scattered shrubland with spinifex grass. Graph (a) reflects habitat selection by all 20 bandicoots. Graph (b) reflects the 10 bandicoots trapped along the central track of the reserve. Graph (c) reflects the 10 bandicoots trapped along the northern track. Positive values represent habitat used more than random and negative values represent habitat used less than random. Asterisks indicate habitats selected significantly more or less than at random (*p* < .05*)

Within the home ranges of all 20 bandicoots (third‐order habitat selection), scattered shrubland with spinifex grass habitat was significantly selected as refuge locations within the diurnal sheltering period, (*p* = 1.06^−8^; Figure 5a), whereas bare understory was significantly avoided (*p* = 6.05^−7^; Table S1). Of the 228 refuges identified, 84% were located under a spinifex hummock. Refuge locations typically had sandy soil with less than 10% overstory cover (Table 4). There were no significant relationships within nocturnal third‐order habitat selection data.

**TABLE 4:** Characteristics of the total refuge sites (*n* = 228) occupied by golden bandicoots when homing during the diurnal period. Percentages are not cumulative for soil type and vegetation overstory as variables could co‐occur

Animal group	Soil			
	Sand (%)	Loam (%)	Clay (%)	Gravel (%)
Central (118)	77.1	39.0	17.8	0.0
Northern (110)	66.4	31.8	29.1	0.9
Total (228)	71.9	35.5	23.2	0.4

	Refuge location			
	Under spinifex clump (%)	Under other grass type (%)	Burrow (adopted or self‐made) (%)	Tree hollow or under fallen branches (%)
Central	92.4	4.2	0.8	2.5
Northern	73.6	4.5	5.5	16.4
Total	83.8	4.4	3.1	9.2

	Location in the landscape: Veg understory			
	Spinifex (>10% veg cover) (%)	Other grass type (>10% veg cover) (%)	Bare (≤10% veg cover) (%)	
Central	95.8	2.5	1.7	
Northern	79.1	19.1	1.8	
Total	87.7	10.5	1.8	

	Location in the landscape: Veg overstory			
	Acacia/mulga (>10% veg cover) (%)	Eucalyptus/mallee (>10% veg cover) (%)	Minimal overstory (≤10% veg overstory) (%)	
Central	21.2	2.5	76.3	
Northern	31.8	13.6	60.0	
Total	26.3	7.9	68.4	

More biologically meaningful trends were detected when we separated datasets associated with bandicoots trapped on the central track from those trapped on the northern track. As per the third‐order habitat selection for all 20 bandicoots, central bandicoots significantly selected for scattered shrubland with spinifex grass habitat during diurnal period (coefficient = 7.92; *p* = 2.34^−7^; Figure 5b), whereas the bare understory was significantly avoided (coefficient = −5.70; *p* = 7.78^−5^). Habitat selection during the nocturnal foraging period was not significantly different from random. Statistical results were similar for northern bandicoots, however, we found positive coefficients for mulga with tuft grass (Figure 5c), which dominates the area in which the animals were trapped (Figure 3), during diurnal and nocturnal periods. The selection of scattered shrubland with spinifex grass during the diurnal period remained significantly positive despite its lack of representation in the immediate area and starkly contrasted the near random use of this habitat during the nocturnal period.

**FIGURE 6:**
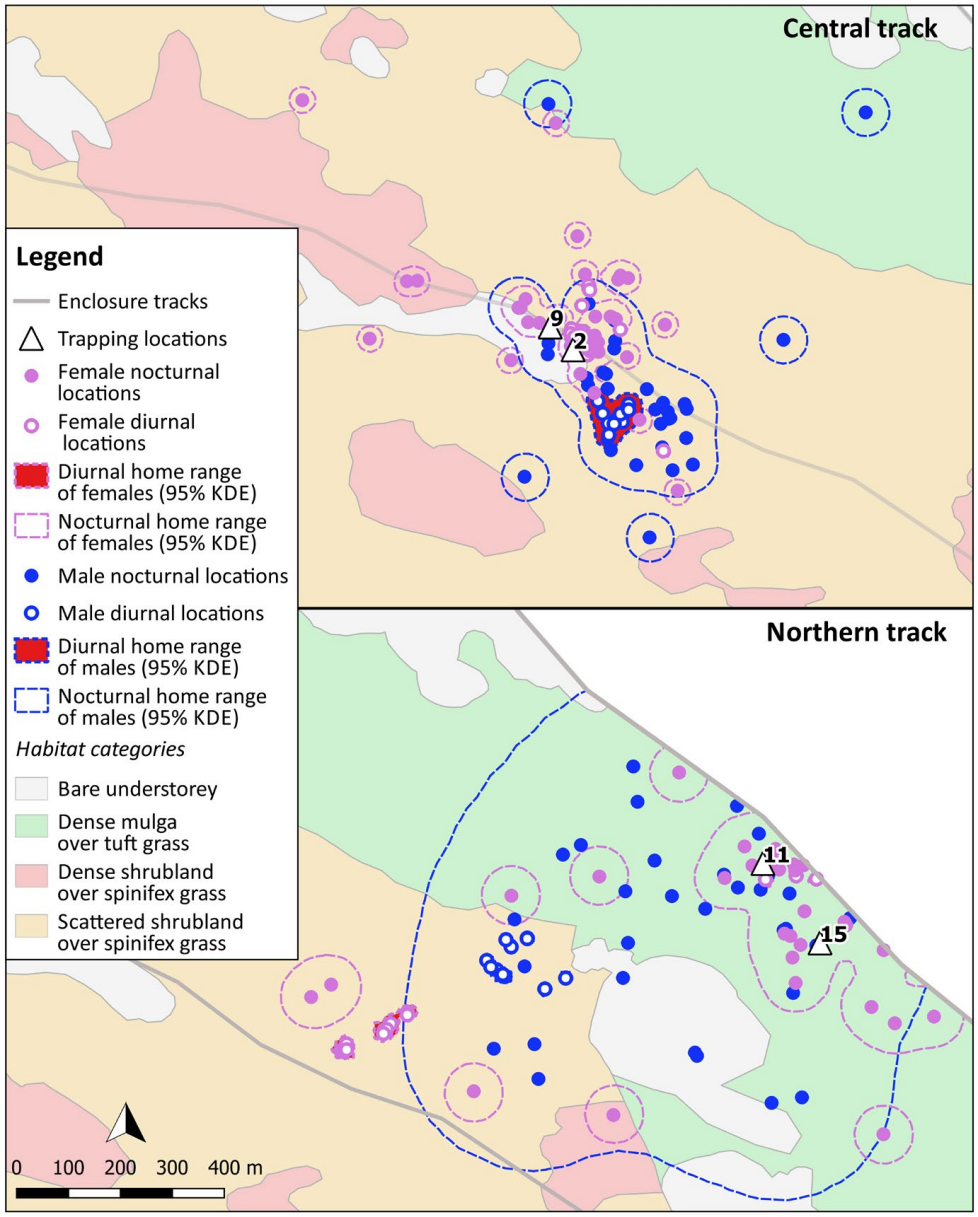
Radiolocations and estimated home‐range sizes (95% KDE) of 4 animals, a male and female from the central track (GB02 and GB09), and a male and female from the northern track (GB11 and GB15)

Trapping location (North or Central track) was not a significant factor in explaining variation in diurnal refuge selection (Table 4); however, trapping locations did appear to influence the selection of nocturnal foraging habitat (Figures 5 and 6). Bandicoots trapped on the northern track selected for dense mulga with tuft grass habitat during the nocturnal foraging period (coefficient = 1.97; *p* = .14), whereas bandicoots trapped on the central track selected for scattered shrubland with spinifex grass habitat (coefficient = 1.78; *p* = .18). The avoidance of dense shrubland over spinifex was seen across all twenty bandicoots.

### 3.4 Radiotracking error

**FIGURE 7:**
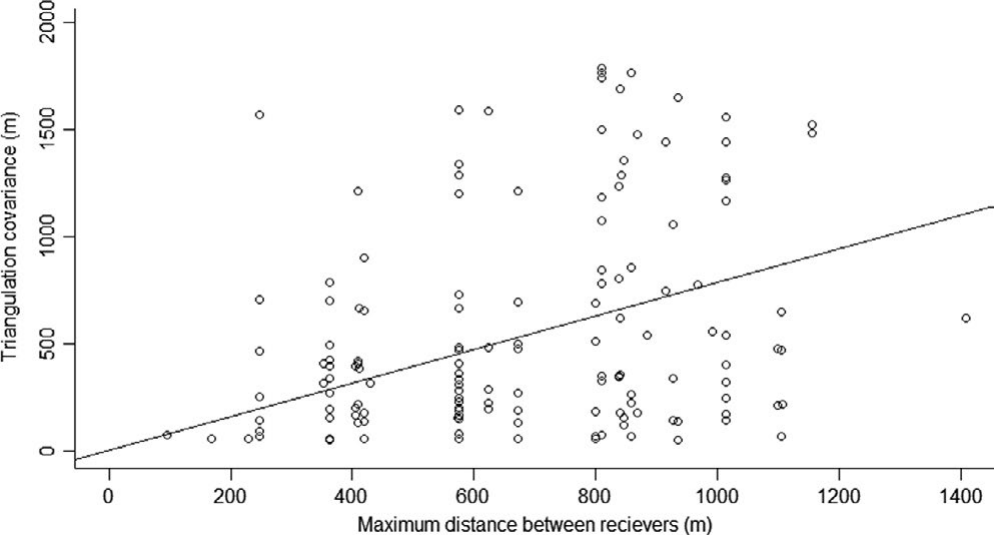
Response of covariance to maximum distance maintained between receivers when radiotracking (*n* = 143)

A significant positive relationship was found between the maximum distance between receiver locations and triangulation covariance of the estimated signal (Table 5; *p* = .01). As seen in Figure 7, covariance significantly increases when receivers are more than 250 m apart. Reference locations were approximately 200 m apart. As expected, there was a negative relationship between the distance between the receiver and the signal and the covariance but decline in signal strength was not a significant explanatory variable of covariance. It is possible that signal reflection off the enclosure fence increased signal covariance. This problem may be reduced by using ≥3 bearings for triangulation (Garrott et al., 1986).

**TABLE 5:** Linear regression results for potential explanatory variables for variation in the estimated covariance of telemetry locations. Covariance (CoVar), MaxRDistS (maximum distance between receiver and signal), MaxRDistR (maximum distance between two receivers), and TotalA (total angle at apex of triangle created by signal and location of two receivers)

Model	Adj *R*²	*F* statistic	DF	Residual *SE*
(CoVar ~ MaxRDistS + MaxRDistR + TotalA − 1)	0.58	66.44	140	486.30

Predictor variables	Coefficients	Estimate Std. Error	t value	Pr(>|t|)
MaxRDistS	−0.1492	0.2746	−0.543	0.5879
MaxRDistR	0.8828	0.3465	2.548	0.0119
TotalA	0.3013	0.891	0.338	0.7357

## 4 DISCUSSION

Our results supported both of our hypotheses: 1) We expected golden bandicoots to select vegetation with an understory of hummock/spinifex grasses for shelter and protection rather than open mulga woodlands and 2) we expected home‐range characteristics to differ between the sexes.

Diurnal refuge locations occurred in scattered shrubland over spinifex significantly more than in proportion to availability for both bandicoots trapped near that habitat (central track) and bandicoots trapped in dense mulga with tuft grass. This is consistent with past radiotracking studies on golden bandicoots (Graham, 1996; Southgate et al., 1996), other bandicoot species, (Chambers & Dickman, 2002), and other small insectivorous marsupials (Bos & Carthew, 2003; Haythornthwaite, 2005).

Spinifex provides refuge from predators, insulation from temperature extremes, and a stable food resource by hosting invertebrate populations (Chambers & Dickman, 2002). Dome‐forming hummocks such as *T. basedowii* are especially insulative (Churchill, 2001) and used by three species of dunnart as shelter, *Sminthopsis psammophila*, *S. ooldea,* and *S. youngsoni* (Riley, 2020). Ground temperatures in the arid zone can be extreme with records of −4.5 to 61°C in the Western Australia Great Victoria Desert (Riley, 2020) and potentially lethal to fauna if appropriate thermally insulative shelter is not available (Kinlaw, 1999). Similar benefits are seen in other vegetation types such as grass trees where dense canopies provide shelter and insulation (Frazer, 2005; Frazer & Petit, 2007; Keiper & Johnson, 2004), whereas, other shelter types, such as fallen logs or ring‐forming spinifex species (e.g., *Triodia* *desertorum*) do not provide the same insulative properties as hummock species, but may be used during milder weather (Riley, 2020). *T. basedowii* is the dominant spinifex species in our study site.

Golden bandicoots are thought to be a polygynous species (Ottewell et al., 2014). Polygynous species have been experimentally shown to exhibit sexually diethic traits such as variation in home‐range size with the promiscuous sex having the larger range (Gaulin & FitzGerald, 1988). Males of most bandicoot species have larger home ranges (Van Dyck & Strahan, 2008). Our results also showed males to have a larger home range than females, providing further evidence that golden bandicoots are polygynous.

The range size and selection of nocturnal foraging habitat among golden bandicoots varied within our study. Central bandicoots generally remained close to their refuge with an average home‐range size half that of northern bandicoots, with few long‐distance foraging forays, similar to activity described for Kimberley mainland bandicoots (Graham, 1996). Northern bandicoots traveled greater distances, usually north from spinifex habitat that provided diurnal refuge toward dense mulga with tuft grass and back. Radiolocations of northern animals are sparser suggesting animals are exhibiting greater maneuverability and expending more energy searching for food.

Animals from the northern part of the enclosure are exhibiting a riskier foraging strategy by traveling further from their primary refuge habitat to forage in more open habitat. This foraging strategy was also seen on Marchinbar Island (Southgate et al., 1996). Greater mean daily movement of individuals is associated with increased risk of mortality in other species (Lohr et al., 2011). Open habitat is considered riskier as predators that hunt visually, such as birds of prey, can detect and capture prey more readily than in dense vegetation (Brown et al., 1988; Meyer & Valone, 1999). On the other hand, prey abundance and diversity can be higher in open habitat for bandicoots (Scott et al., 1999) and dasyurid species (Fisher & Dickman, 1993).

We propose two hypotheses as to why a riskier foraging strategy is exhibited by northern animals. First, scattered shrubland with spinifex grass may be a more resource‐rich environment than dense mulga with tuft grass. Since bandicoot home range is reportedly negatively correlated with food abundance (Broughton & Dickman, 1991), northern bandicoots need to travel further than central bandicoots to attain sufficient food resources. Second, a higher density of animals and associated intraspecific competition and territorial behaviors may be interacting with the resource‐rich spinifex to constrain bandicoot home ranges (Schradin et al., 2010). Territorial behaviors, however, have not been observed in closely related southern brown bandicoots (*Isoodon* *fusciventer*) (Broughton & Dickman, 1991; Thavornkanlapachai et al., 2021; Travouillon & Phillips, 2018).

Unlikely, alternative hypotheses are that the energetic reward of food available in dense mulga with tuft grass may outweigh the risk of predation and be encouraging bandicoots whose home range is near the ecotone between the habitats to forage widely. Or bandicoots, like many other species, may require a habitat mosaic to maximize foraging efficiency and increase rates of reproduction and survival (Law & Dickman, 1998), although a need for a habitat mosaic may explain why the more extreme habitat types of dense shrubland with spinifex and bare understory were used less than expected given their availability. Interspecific competition was deemed unlikely as the only potentially competing species are brush‐tailed mulgara (*Dasycercus blythi*), who forage for invertebrates in topsoil (Molyneux et al., 2018; Pavey et al., 2018) while bandicoots consume subfossorial invertebrates (Southgate et al., 1996).

Intraspecific competition and resultant emigration may also explain why the density of golden bandicoots in the Matuwa enclosure is approximately one quarter of the density recorded in the founder population on Barrow Island which was 1.65 to 1.72 bandicoots per hectare (Teale, 2013). As in striped mice (*Rhabdomys pumilio*) (Schradin et al., 2010) high densities of animals and associated intraspecific competition may limit a bandicoots’ home range and hence access to resources, which may manifest as reduced bodyweight, survival, or reproductive output. When golden bandicoots were translocated from Barrow Island to Matuwa, there was a sudden increase in individuals mass by 28%–34% for males and females, respectively (Dunlop et al., 2018). A similar increase in mass occurred when bandicoots were translocated from the Matuwa enclosure to the neighboring open landscape (Blythman et al., 2020) suggesting the translocated individuals were released from an environment with limited resources. Golden bandicoots are capable of emigrating through the fenced enclosure despite the use of fine mesh (40 mm “rabbit wire”) and have been observed surviving on the open landscape in very low densities (Blythman et al., 2020) and have appeared as prey items in dingo scats outside of the fenced area (Wysong et al., 2019). If bandicoots are regularly dispersing through the fence of the enclosure, then we would expect the density of the remaining population to be lower than a closed system (Barrow Island) or carrying capacity.

We selected the simpler *PhiD* model on subset CMR data to predict the density and abundance of golden bandicoots in the Matuwa enclosure. The *Global* model predicted wide fluctuations in bandicoot abundance over the last 10 years (Supplementary Information S1). The modeling package *openCR* is a new but advanced modeling system that allows us to use spatially explicit capture–recapture data analysis for open populations. All other modeling systems assume a population is closed, which is frequently a fundamentally flawed assumption. Limitations of *openCR* include an inability to perform goodness‐of‐fit tests, AIC model ranking, or any adjustments for overdispersion of data (Efford, 2019). This limits our ability to determine statistically whether the simpler model presented here is better than the *Global* model.

### 4.1 Management implications

Our research has demonstrated that in the central Australian arid zone, golden bandicoots will use a diverse array of habitat types but appear to select scattered shrubland with spinifex grass, probably because of the insulative properties of hummock forming spinifex. Until further research confirms or refutes our results, ideally with a larger number of female bandicoots, future translocation proposals for golden bandicoots should demonstrate that their selected translocation sites contain considerable quantities of dome‐forming spinifex. Additional studies into the diet and body condition of golden bandicoots as it relates to habitat selection would also be beneficial as there is some suggestion that small mammal species that occupy dry habitats are dietary generalists but habitat specialists (Braithwaite & Gullan, 1978). A better understanding of this relationship in the context of Australian fauna could be informative in planning translocations. We also recommend that future research ascertain the rate of golden bandicoot dispersal through fences as unmeasured loss of animals to emigration will affect managers’ interpretation of population parameters within fenced enclosures.

## CONFLICT OF INTEREST

None declared.

## AUTHOR CONTRIBUTIONS

**Cheryl A. Lohr:** Formal analysis (lead); Methodology (lead); Software (equal); Supervision (lead); Writing‐original draft (lead); Writing‐review & editing (equal). **Kristen Nilsson:** Data curation (lead); Formal analysis (equal); Software (equal); Writing‐original draft (equal). **Colleen Sims:** Conceptualization (equal); Data curation (equal); Methodology (equal); Writing‐review & editing (equal). **Judy Dunlop:** Conceptualization (equal); Visualization (equal); Writing‐review & editing (equal). **Michael T. Lohr:** Conceptualization (equal); Formal analysis (supporting); Writing‐review & editing (equal).

## Supporting information

Figure S1

Supplementary Material

## Data Availability

Data used in this study are available for download in Dryad ("Golden bandicoot raw tracking data"; https://doi.org/10.5061/dryad.0k6djhb0r).
