# Supplementary figures and images for: Habitat selection by vulnerable golden bandicoots in the arid zone
Source: Ecol Evol. 2021 Jul 8;11(15):10644–58. doi: 10.1002/ece3.7875 (PMC8328459; doi:10.1002/ece3.7875)

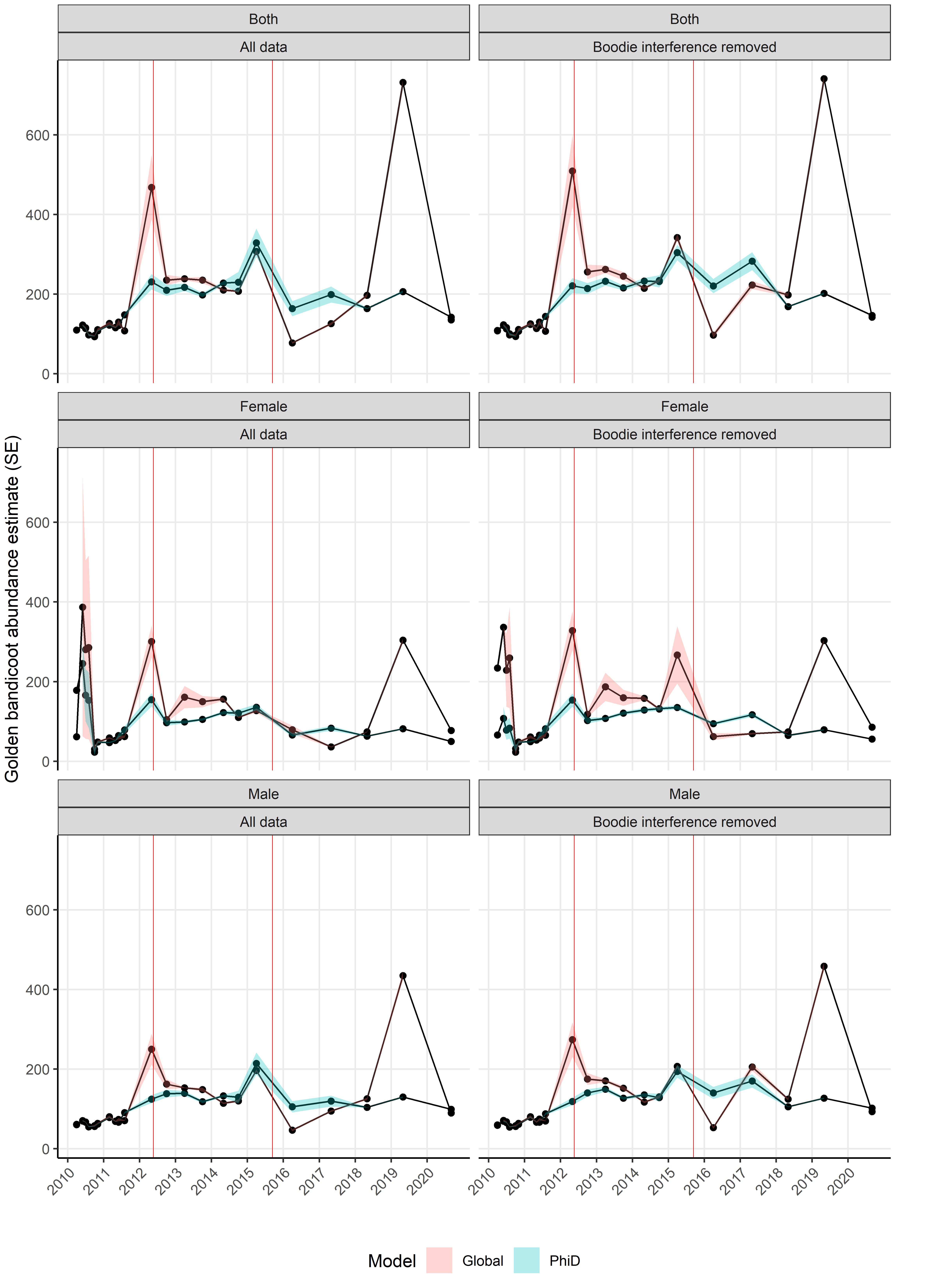

Supplement: Supplementary file 1 — Figure S1 [file ECE3-11-10644-s001.tiff]
