# Supplementary material for: Habitat selection by vulnerable golden bandicoots in the arid zone
Source: Ecol Evol. 2021 Jul 8;11(15):10644–58. doi: 10.1002/ece3.7875 (PMC8328459; doi:10.1002/ece3.7875)
Supplement: Supplementary file 2 — Supplementary Material [file ECE3-11-10644-s002.docx]

# Appendix


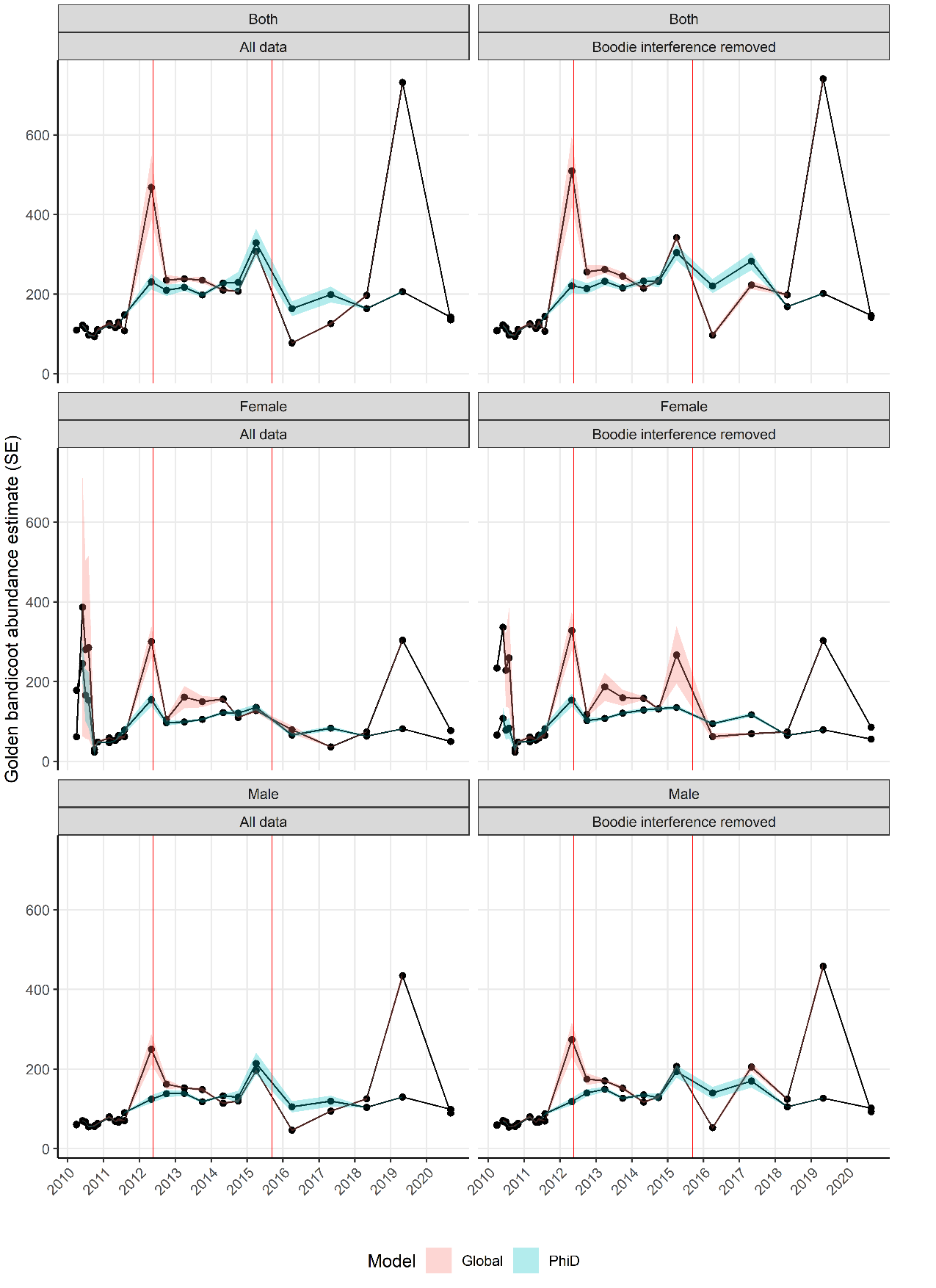


#### Figure S1: Average abundance estimates for golden bandicoots in the fenced enclosure at Matuwa generated by global models (sigma ~ t, lambda0 ~ t, phi ~ t, D ~ t) and PhiD models (sigma ~ 1, lambda0 ~ 1, phi ~ t, D ~ t) in the R package ‘openCR’ for all golden bandicoots, female bandicoots and males. Models in the left column estimated using all capture data whereas models in the right column are estimated from a subset of capture data that excludes any traps that captured alternative species.

#### Table S1: Results of linear mixed effects models comparing second order habitat selection, and third order habitat selection for nocturnal and diurnal habitat points with sex and trapping location as factors. Analysis performed using R package lme4 (Bates et al. 2014).

| **Second order habitat selection** | | | | | |
| --- | --- | --- | --- | --- | --- |
| Linear mixed model fit by REML. t-tests use Satterthwaite's method ['lmerModLmerTest']  Formula: Second order ~ Habitat + Trapped + Sex + Habitat: Trapped + (1 \| Bandicoot ID) | | | | | |
| Random effects | Variance | SD |  |  |  |
| ID | 5.71^-21^ | 7.55^-11^ |  |  |  |
| Residual | 7.11 | 2.66 |  |  |  |
| Fixed effects | Estimate | SE | Df | T | P |
| Intercept | 6.00^-12^ | 0.92 | 89.00 | 0.00 | 1.00 |
| Bare understorey | 0.96 | 1.19 | 89.00 | 0.81 | 0.42 |
| Dense shrubland with spinifex | -0.49 | 1.19 | 89.00 | -0.42 | 0.68 |
| Dense mulga with tuft grass | -2.69 | 1.19 | 89.00 | -2.25 | 0.03 |
| Scattered shrubland with spinifex grass | 2.22 | 1.19 | 89.00 | 1.86 | 0.07 |
| Trapped - Northern | 1.99^-12^ | 1.20 | 89.00 | 0.00 | 1.00 |
| Sex – Male | -1.00^-11^ | 0.60 | 89.00 | 0.00 | 1.00 |
| Bare understorey: Trapped - Northern | -0.69 | 1.68 | 89.00 | -0.41 | 0.69 |
| Dense shrubland with spinifex: Trapped - Northern | -4.46 | 1.68 | 89.00 | -2.65 | 9.65^-3^ |
| Dense mulga with tuft grass: Trapped - Northern | 5.82 | 1.68 | 89.00 | 3.45 | 8.50^-4^ |
| Scattered shrubland with spinifex grass: Trapped - Northern | -0.68 | 1.68 | 89.00 | -0.40 | 0.69 |
|  |  |  |  |  |  |
| **Third order habitat selection with nocturnal points** | | | | | |
| Linear mixed model fit by REML. t-tests use Satterthwaite's method ['lmerModLmerTest']  Formula: Third order.nocturnal ~ Habitat + (1 \| ID) | | | | | |
| Random effects | Variance | SD |  |  |  |
| ID | 1.77^-20^ | 1.33^-10^ |  |  |  |
| Residual | 8.91 | 2.99 |  |  |  |
| Fixed effects | Estimate | SE | Df | T | P |
| Intercept | -1.33^-17^ | 0.67 | 95.00 | 0.00 | 1.00 |
| Bare understorey | 0.08 | 0.94 | 95.00 | 0.09 | 0.93 |
| Dense shrubland with spinifex | -1.35 | 0.94 | 95.00 | -1.43 | 0.16 |
| Dense mulga with tuft grass | 0.45 | 0.94 | 95.00 | 0.48 | 0.64 |
| Scattered shrubland with spinifex grass | 0.82 | 0.94 | 95.00 | 0.88 | 0.39 |
|  |  |  |  |  |  |
| **Third order habitat selection with diurnal points** | | | | | |
| Linear mixed model fit by REML. t-tests use Satterthwaite's method ['lmerModLmerTest']  Formula: Third order.diurnal ~ Habitat + (1 \| ID) | | | | | |
| Random effects | Variance | SD |  |  |  |
| ID | 1.36^-20^ | 1.17^-10^ |  |  |  |
| Residual | 12.42 | 3.52 |  |  |  |
| Fixed effects | Estimate | SE | Df | T | P |
| Intercept | -1.04^-15^ | 0.79 | 95.00 | 0.00 | 1.00 |
| Bare understorey | -5.96 | 1.11 | 95.00 | -5.35 | 6.05^-7^ |
| Dense shrubland with spinifex | -1.08 | 1.11 | 95.00 | -0.97 | 0.33 |
| Dense mulga with tuft grass | 5.95^-2^ | 1.11 | 95.00 | 0.05 | 0.96 |
| Scattered shrubland with spinifex grass | 6.99 | 1.11 | 95.00 | 6.27 | 1.06^-8^ |

#### Table S2: AIC model ranking for the four best models that explain variation in signal covariance (CoVar) given variation in MaxRDistS (Maximum distance between receiver and signal), MaxRDistR (Maximum distance between two receivers), and TotalA (Total angle at apex of triangle created by signal and location of two receivers). Models used were; CoVar~MaxRDistS+MaxRDistR-1 (m1), CoVar~MaxRDistS+MaxRDistR+TotalA (m2), CoVar~MaxRDistR-1 (m3), CoVar~MaxRDistS+MaxRDistR+TotalA-1 (m4).

| Model | Adj R² | F statistic | DF | Residual SE | K | AICc | ∆AICc | Model likelihood | AICc weight | Log likelihood |
| --- | --- | --- | --- | --- | --- | --- | --- | --- | --- | --- |
| m1 | 0.09 | 5.68 | 139 | 476.20 | 5 | 2175.60 | 0.00 | 1.00 | 0.57 | -1082.58 |
| m2 | 0.58 | 199.90 | 142 | 484.50 | 2 | 2177.26 | 1.66 | 0.44 | 0.25 | -1086.59 |
| m3 | 0.58 | 100.20 | 141 | 484.80 | 3 | 2178.51 | 2.91 | 0.23 | 0.13 | -1086.17 |
| m4 | 0.58 | 66.44 | 140 | 486.30 | 4 | 2180.51 | 4.91 | 0.09 | 0.05 | -1086.11 |

#### Table S3. Summary information for the 20 golden bandicoots that were radio-tracked within the Matuwa fenced enclosure.

| Individual | Sex | Survey period | Tracking period (days) | Telemetry positions | Triangulations | Mean co variance (m) of triangulations (≥3 bearings) | Biangulations | No. of triangulations and biangulations considered errors and removed by LOAS | No. of locations manually removed by falling outside the enclosure | Fixed 95% KDE (ha) | MCP (ha) |
| --- | --- | --- | --- | --- | --- | --- | --- | --- | --- | --- | --- |
| GB01 | M | Diurnal |  | 13 |  |  |  |  |  | 6.26 | 6.21 |
|  |  | Nocturnal |  | 34 | 16 | 1275.70 | 18 | 3 | 1 | 27.95 | 66.56 |
|  |  | Total | 37 | 47 |  |  |  |  |  | 26.10 | 66.56 |
| GB02 | M | Diurnal |  | 12 |  |  |  |  |  | 2.47 | 0.22 |
|  |  | Nocturnal |  | 36 | 17 | 375.94 | 19 | 2 | 1 | 15.71 | 38.10 |
|  |  | Total | 39 | 48 |  |  |  |  |  | 8.12 | 38.10 |
| GB03 | M | Diurnal |  | 5 |  |  |  |  |  | 11.71 | 2.47 |
|  |  | Nocturnal |  | 13 | 10 | 11242.81 | 3 | 0 | 0 | 112.75 | 26.65 |
|  |  | Total | 19 | 19 |  |  |  |  |  | 3.04 | 26.65 |
| GB04 | M | Diurnal |  | 13 |  |  |  |  |  | 2.70 | 0.48 |
|  |  | Nocturnal |  | 32 | 19 | 1589.42 | 26 | 4 | 0 | 44.37 | 16.86 |
|  |  | Total | 39 | 45 |  |  |  |  |  | 15.24 | 16.86 |
| GB05 | F | Diurnal |  | 14 |  |  |  |  |  | 3.73 | 0.86 |
|  |  | Nocturnal |  | 29 | 16 | 36542.10 | 13 | 4 | 1 | 93.51 | 195.21 |
|  |  | Total | 39 | 43 |  |  |  |  |  | 16.38 | **195.21** |
| GB06 | F | Diurnal |  | 16 |  |  |  |  |  | 2.63 | 0.48 |
|  |  | Nocturnal |  | 33 | 16 | 502.14 | 17 | 3 | 0 | 22.79 | 19.36 |
|  |  | Total | 39 | 49 |  |  |  |  |  | 7.61 | 19.36 |
|  |  |  |  |  |  |  |  |  |  |  |  |
| GB07 | M | Diurnal |  | 13 |  |  |  |  |  | 7.51 | 6.91 |
|  |  | Nocturnal |  | 24 | 15 | 958.35 | 9 | 3 | 0 | 24.69 | 13.45 |
|  |  | Total | 39 | 37 |  |  |  |  |  | 19.95 | 27.63 |
| GB08 | M | Diurnal |  | 16 |  |  |  |  |  | 6.03 | 4.33 |
|  |  | Nocturnal |  | 24 | 13 | 51071.03 | 11 | 7 | 2 | 94.29 | 61.45 |
|  |  | Total | 39 | 40 |  |  |  |  |  | 17.80 | 61.45 |
| GB09 | F | Diurnal |  | 15 |  |  |  |  |  | 2.21 | 1.59 |
|  |  | Nocturnal |  | 37 | 25 | 348.77 | 12 | 1 | 0 | 11.36 | 31.43 |
|  |  | Total | 39 | 52 |  |  |  |  |  | 5.43 | 31.43 |
| GB10 | F | Diurnal |  | 1 |  |  |  |  |  | - | - |
|  |  | Nocturnal |  | 7 | 4 | 1421.09 | 3 | 2 | 0 | 21.59 | 67.27 |
|  |  | Total | 5 | 8 |  |  |  |  |  | 21.13 | 67.27 |
| GB11 | M | Diurnal |  | 14 |  |  |  |  |  | 2.36 | 0.85 |
|  |  | Nocturnal |  | 31 | 19 | 1234.13 | 12 | 2 | 8 | 66.70 | 31.33 |
|  |  | Total | 37 | 45 |  |  |  |  |  | 8.23 | 31.84 |
| GB12 | M | Diurnal |  | 15 |  |  |  |  |  | 1.69 | 1.60 |
|  |  | Nocturnal |  | 19 | 15 | 202.99 | 4 | 4 | 1 | 26.39 | 26.23 |
|  |  | Total | 39 | 34 |  |  |  |  |  | 3.09 | 29.72 |
| GB13 | F | Diurnal |  | 12 |  |  |  |  |  | 1.97 | 0.26 |
|  |  | Nocturnal |  | 28 | 16 | 11509.60 | 12 | 4 | 7 | 37.88 | 46.76 |
|  |  | Total | 36 | 40 |  |  |  |  |  | 3.35 | 46.76 |
| GB14 | M | Diurnal |  | 4 |  |  |  |  |  | 3.99 | 1.83 |
|  |  | Nocturnal |  | 11 | 7 | 3774.62 | 4 | 1 | 0 | 108.34 | 50.55 |
|  |  | Total | 14 | 16 |  |  |  |  |  | 113.36 | 51.93 |
| GB15 | F | Diurnal |  | 18 |  |  |  |  |  | 4.32 | 17.86 |
|  |  | Nocturnal |  | 29 | 18 | 3924.24 | 11 | 3 | 11 | 20.69 | 49.09 |
|  |  | Total | 38 | 47 |  |  |  |  |  | 4.69 | 53.40 |
| GB16 | M | Diurnal |  | 14 |  |  |  |  |  | 3.66 | 5.90 |
|  |  | Nocturnal |  | 27 | 19 | 25880.06 | 8 | 6 | 8 | 159.43 | 128.01 |
|  |  | Total | 37 | 41 |  |  |  |  |  | 11.90 | 128.01 |
| GB17 | M | Diurnal |  | 14 |  |  |  |  |  | 4.85 | 6.21 |
|  |  | Nocturnal |  | 27 | 18 | 3803.94 | 9 | 4 | 7 | 25.44 | 55.47 |
|  |  | Total | 37 | 41 |  |  |  |  |  | 6.73 | 55.47 |
| GB18 | M | Diurnal |  | 12 |  |  |  |  |  | 3.27 | 3.61 |
|  |  | Nocturnal |  | 24 | 13 | 1209.28 | 11 | 2 | 4 | 79.82 | 98.79 |
|  |  | Total | 37 | 36 |  |  |  |  |  | 12.08 | 98.79 |
| GB19 | M | Diurnal |  | 4 |  |  |  |  |  | 2.15 | 0.33 |
|  |  | Nocturnal |  | 5 | 3 | 13074.79 | 2 | 1 | 2 | 279.89 | 30.54 |
|  |  | Total | 14 | 9 |  |  |  |  |  | 3.37 | 35.63 |
| GB20 | M | Diurnal |  | 3 |  |  |  |  |  | 59.68 | 2.39 |
|  |  | Nocturnal |  | 5 | 3 | 963.67 | 2 | 0 | 0 | 120.98 | 9.58 |
|  |  | Total | 5 | 8 |  |  |  |  |  | 53.95 | **12.36** |
| Summary |  | Total locations |  | 703 | 282 | Mean: 8422.74 (±2599.71) | 193 | 56 | 53 |  |  |
